# (*E*)-3-(4-Chloro­phen­yl)-2-phenyl­prop-2-enoic acid

**DOI:** 10.1107/S1600536809021904

**Published:** 2009-06-13

**Authors:** Sadiq-ur-Rehman, Saqib Ali, Saira Shahzadi, Masood Parvez

**Affiliations:** a Department of Chemistry, University of Azad Jammu and Kashmir, Muzaffarabad 13100, Pakistan; b Department of Chemistry, Quaid-i-Azam University, Islamabad 45320, Pakistan; c Department of Chemistry, GC University, Faisalabad, Pakistan; d Department of Chemistry, The University of Calgary, 2500 University Drive NW, Calgary, Alberta, Canada T2N 1N4

## Abstract

In the title mol­ecule, C_15_H_11_ClO_2_, the mean planes of the benzene and phenyl rings are inclined at 69.06 (11)° with respect to each other. The crystal structure is stablized by strong inter­molecular O—H⋯O hydrogen bonds between the acid groups of pairs of mol­ecules related by inversion centers.

## Related literature

For background information, see: Canty & Van Koten (1995 ▶). For a related structure, see: Sadiq-ur-Rehman *et al.* (2006 ▶). For a description of the Cambridge Structural Database, see: Allen (2002 ▶).
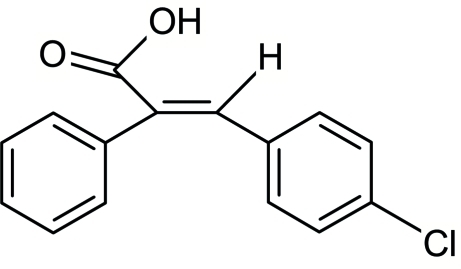


## Experimental

### 

#### Crystal data

C_15_H_11_ClO_2_
*M* *_r_* = 258.69
Monoclinic, 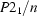

*a* = 14.405 (3) Å
*b* = 5.733 (9) Å
*c* = 15.416 (9) Å
β = 100.72 (3)°
*V* = 1251 (2) Å^3^
*Z* = 4
Mo *K*α radiation
μ = 0.30 mm^−1^
*T* = 173 K
0.16 × 0.10 × 0.04 mm

#### Data collection

Nonius KappaCCD diffractometer
Absorption correction: multi-scan (*SORTAV*; Blessing, 1997 ▶) *T*_min_ = 0.954, *T*_max_ = 0.988
10074 measured reflections
2860 independent reflections
1431 reflections with *I* > 2σ(*I*)
*R*_int_ = 0.100

#### Refinement

*R*[*F* ^2^ > 2σ(*F* ^2^)] = 0.047
*wR*(*F* ^2^) = 0.130
*S* = 0.96
2860 reflections
164 parameters
H-atom parameters constrained
Δρ_max_ = 0.22 e Å^−3^
Δρ_min_ = −0.33 e Å^−3^

### 

Data collection: *COLLECT* (Hooft, 1998 ▶); cell refinement: *DENZO* (Otwinowski & Minor, 1997 ▶); data reduction: *SCALEPACK* (Otwinowski & Minor, 1997 ▶); program(s) used to solve structure: *SHELXS97* (Sheldrick, 2008 ▶); program(s) used to refine structure: *SHELXL97* (Sheldrick, 2008 ▶); molecular graphics: *ORTEP-3 for Windows* (Farrugia, 1997 ▶); software used to prepare material for publication: *SHELXTL* (Sheldrick, 2008 ▶).

## Supplementary Material

Crystal structure: contains datablocks global, I. DOI: 10.1107/S1600536809021904/lh2835sup1.cif

Structure factors: contains datablocks I. DOI: 10.1107/S1600536809021904/lh2835Isup2.hkl

Additional supplementary materials:  crystallographic information; 3D view; checkCIF report

## Figures and Tables

**Table 1:** Hydrogen-bond geometry (Å, °)

*D*—H⋯*A*	*D*—H	H⋯*A*	*D*⋯*A*	*D*—H⋯*A*
O1—H1⋯O2^i^	0.84	1.82	2.658 (3)	177

Symmetry code: (i) 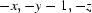.

**Fig. 1.**
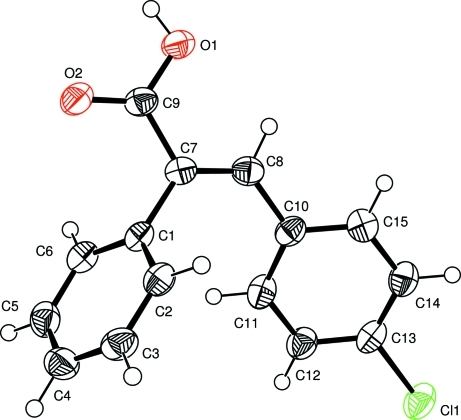
ORTEP-3 (Farrugia, 1997) drawing of (I) with displacement ellipsoids plotted at 50% probability level.

**Fig. 2.**
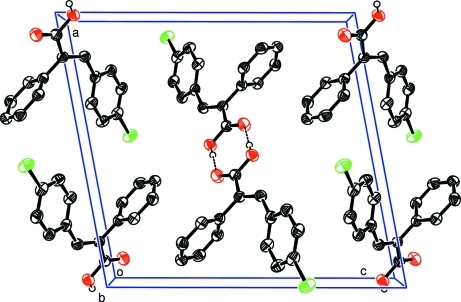
Unit cell packing of (I) showing hydrogen bonding (dashed lines); H-atoms not involved in hydrogen bonding have been excluded for clarity.

## supplementary crystallographic information

### Comment

Effort has been devoted to self assembly of organic and inorganic molecules in solid state because it extends a range of new solids with desirable physical and chemical properties (Canty & Van Koten, 1995). We report in this paper the crystal structure of the title compound (I) which has been synthesized in our laboratory.

The molecular structure of (I) is presented in Fig. 1. The benzene and phenyl rings are oriented at 69.06 (11)° with respect to each other. Molecules related by inversion ceneters form dimers *via* hydrogen bonds (Fig. 2); details of hydrogen bonding geometry have been given in Table 1. The molecular dimensions are normal (Allen, 2002). The cystal structure of a closely related compound has been previously reported from our laboratory (Sadiq-ur-Rehman *et al.*, 2006).

### Experimental

A mixture of the phenylacetic acid (0.15 mol), *p*-chlorobenzaldehyde (0.15 mol), anhydrous K_2_CO_3_ (0.095 mol) and acetic anhydride (0.38 mol) was slowly raised to the temperature 353–373 K and maintained for 24 h. To a hot solution were added, 200 ml of H_2_O and 100 ml of 10% HCl. The mixture was stirred at room temperature for 2 h and filtered. The solid mass obtained was recrystallized from commercial ethanol. Colorless crystals suitable for crystallographic study were obtained after three weeks.

### Refinement

All the H-atoms were visible in the difference Fourier maps, they were included in the refinements at geometrically idealized positions with C—H and O—H distances = 0.95 and 0.84 Å, respectively, and *U*_iso_ = 1.5 and 1.2 times *U*_eq_ of the parent O and C-atoms respectively. The final difference map was free of chemically significant features.

### Crystal data

**Table:**

C_15_H_11_ClO_2_	*F*(000) = 536
*M**_r_* = 258.69	*D*_x_ = 1.374 Mg m^−^^3^
Monoclinic, *P*2_1_/*n*	Mo *K*α radiation, λ = 0.71073 Å
Hall symbol: -P 2yn	Cell parameters from 10074 reflections
*a* = 14.405 (3) Å	θ = 1.8–27.5°
*b* = 5.733 (9) Å	µ = 0.30 mm^−^^1^
*c* = 15.416 (9) Å	*T* = 173 K
β = 100.72 (3)°	Block, colourless
*V* = 1251 (2) Å^3^	0.16 × 0.10 × 0.04 mm
*Z* = 4	

### Data collection

**Table:**

Nonius KappaCCD diffractometer	2860 independent reflections
Radiation source: fine-focus sealed tube	1431 reflections with *I* > 2σ(*I*)
graphite	*R*_int_ = 0.100
φ and ω scans	θ_max_ = 27.5°, θ_min_ = 1.8°
Absorption correction: multi-scan (*SORTAV*; Blessing, 1997)	*h* = −18→18
*T*_min_ = 0.954, *T*_max_ = 0.988	*k* = −7→7
10074 measured reflections	*l* = −19→19

### Refinement

**Table:**

Refinement on *F*^2^	Primary atom site location: structure-invariant direct methods
Least-squares matrix: full	Secondary atom site location: difference Fourier map
*R*[*F*^2^ > 2σ(*F*^2^)] = 0.047	Hydrogen site location: inferred from neighbouring sites
*wR*(*F*^2^) = 0.130	H-atom parameters constrained
*S* = 0.96	*w* = 1/[σ^2^(*F*_o_^2^) + (0.06*P*)^2^] where *P* = (*F*_o_^2^ + 2*F*_c_^2^)/3
2860 reflections	(Δ/σ)_max_ < 0.001
164 parameters	Δρ_max_ = 0.22 e Å^−^^3^
0 restraints	Δρ_min_ = −0.33 e Å^−^^3^

### Special details

**Table:**

Geometry. All e.s.d.'s (except the e.s.d. in the dihedral angle between two l.s. planes) are estimated using the full covariance matrix. The cell e.s.d.'s are taken into account individually in the estimation of e.s.d.'s in distances, angles and torsion angles; correlations between e.s.d.'s in cell parameters are only used when they are defined by crystal symmetry. An approximate (isotropic) treatment of cell e.s.d.'s is used for estimating e.s.d.'s involving l.s. planes.
Refinement. Refinement of *F*^2^ against ALL reflections. The weighted *R*-factor *wR* and goodness of fit *S* are based on *F*^2^, conventional *R*-factors *R* are based on *F*, with *F* set to zero for negative *F*^2^. The threshold expression of *F*^2^ > σ(*F*^2^) is used only for calculating *R*-factors(gt) *etc*. and is not relevant to the choice of reflections for refinement. *R*-factors based on *F*^2^ are statistically about twice as large as those based on *F*, and *R*- factors based on ALL data will be even larger.

### Fractional atomic coordinates and isotropic or equivalent isotropic displacement parameters (Å^2^)

**Table:**

	*x*	*y*	*z*	*U*_iso_*/*U*_eq_	
Cl1	0.44668 (5)	0.75005 (11)	−0.15436 (5)	0.0494 (2)	
O1	0.01817 (12)	−0.2417 (3)	−0.06692 (11)	0.0418 (5)	
H1	−0.0181	−0.3550	−0.0639	0.063*	
O2	0.09332 (13)	−0.3917 (3)	0.06070 (11)	0.0464 (5)	
C1	0.24216 (16)	−0.0735 (4)	0.07913 (14)	0.0301 (6)	
C2	0.25291 (17)	0.1160 (4)	0.13702 (15)	0.0331 (6)	
H2	0.2109	0.2448	0.1257	0.040*	
C3	0.32420 (18)	0.1175 (4)	0.21066 (16)	0.0390 (6)	
H3	0.3305	0.2456	0.2504	0.047*	
C4	0.38652 (18)	−0.0687 (5)	0.22627 (16)	0.0406 (7)	
H4	0.4364	−0.0668	0.2762	0.049*	
C5	0.37624 (18)	−0.2559 (5)	0.16958 (17)	0.0400 (6)	
H5	0.4191	−0.3830	0.1806	0.048*	
C6	0.30376 (17)	−0.2607 (4)	0.09650 (16)	0.0337 (6)	
H6	0.2963	−0.3922	0.0583	0.040*	
C7	0.16421 (16)	−0.0712 (4)	0.00023 (15)	0.0306 (6)	
C8	0.15911 (17)	0.0761 (4)	−0.06802 (15)	0.0339 (6)	
H8	0.1017	0.0727	−0.1100	0.041*	
C9	0.08865 (17)	−0.2473 (4)	0.00101 (16)	0.0339 (6)	
C10	0.23076 (17)	0.2420 (4)	−0.08639 (15)	0.0323 (6)	
C11	0.32781 (18)	0.2055 (4)	−0.05820 (17)	0.0373 (6)	
H11	0.3488	0.0719	−0.0236	0.045*	
C12	0.39343 (19)	0.3591 (4)	−0.07955 (17)	0.0390 (6)	
H12	0.4590	0.3313	−0.0602	0.047*	
C13	0.36306 (18)	0.5541 (4)	−0.12935 (16)	0.0370 (6)	
C14	0.26819 (18)	0.5965 (4)	−0.15954 (16)	0.0388 (6)	
H14	0.2480	0.7306	−0.1941	0.047*	
C15	0.20296 (18)	0.4382 (4)	−0.13813 (15)	0.0363 (6)	
H15	0.1376	0.4644	−0.1594	0.044*	

### Atomic displacement parameters (Å^2^)

**Table:**

	*U*^11^	*U*^22^	*U*^33^	*U*^12^	*U*^13^	*U*^23^
Cl1	0.0473 (4)	0.0393 (4)	0.0664 (5)	−0.0059 (3)	0.0232 (3)	0.0088 (4)
O1	0.0376 (11)	0.0454 (11)	0.0395 (10)	−0.0145 (9)	−0.0006 (8)	0.0038 (9)
O2	0.0481 (12)	0.0473 (12)	0.0414 (11)	−0.0173 (9)	0.0016 (8)	0.0092 (10)
C1	0.0326 (14)	0.0279 (13)	0.0315 (13)	−0.0053 (11)	0.0109 (11)	0.0003 (11)
C2	0.0369 (15)	0.0277 (14)	0.0361 (14)	−0.0018 (11)	0.0101 (12)	0.0001 (11)
C3	0.0445 (17)	0.0379 (15)	0.0346 (15)	−0.0090 (13)	0.0076 (13)	−0.0065 (12)
C4	0.0342 (15)	0.0536 (17)	0.0336 (14)	−0.0064 (13)	0.0052 (12)	0.0052 (14)
C5	0.0358 (16)	0.0398 (15)	0.0465 (16)	0.0048 (13)	0.0130 (13)	0.0108 (14)
C6	0.0370 (15)	0.0295 (13)	0.0365 (14)	−0.0043 (12)	0.0118 (12)	−0.0020 (12)
C7	0.0293 (14)	0.0312 (14)	0.0315 (13)	−0.0035 (11)	0.0063 (11)	−0.0043 (12)
C8	0.0320 (15)	0.0350 (14)	0.0345 (14)	−0.0015 (11)	0.0053 (11)	−0.0045 (12)
C9	0.0332 (14)	0.0351 (14)	0.0329 (14)	−0.0051 (12)	0.0052 (11)	−0.0046 (13)
C10	0.0383 (15)	0.0309 (13)	0.0287 (13)	−0.0054 (12)	0.0091 (11)	−0.0039 (11)
C11	0.0404 (16)	0.0316 (15)	0.0423 (15)	0.0012 (11)	0.0141 (12)	0.0038 (11)
C12	0.0360 (16)	0.0362 (14)	0.0472 (16)	−0.0005 (12)	0.0143 (12)	0.0036 (13)
C13	0.0440 (17)	0.0283 (13)	0.0425 (15)	−0.0059 (12)	0.0178 (12)	−0.0019 (12)
C14	0.0455 (18)	0.0338 (15)	0.0401 (14)	0.0008 (12)	0.0157 (13)	0.0052 (12)
C15	0.0369 (15)	0.0389 (15)	0.0339 (14)	0.0030 (12)	0.0090 (11)	−0.0002 (12)

### Geometric parameters (Å, °)

**Table:**

Cl1—C13	1.742 (3)	C6—H6	0.9500
O1—C9	1.316 (3)	C7—C8	1.340 (3)
O1—H1	0.8400	C7—C9	1.486 (3)
O2—C9	1.230 (3)	C8—C10	1.469 (3)
C1—C6	1.386 (3)	C8—H8	0.9500
C1—C2	1.396 (3)	C10—C15	1.393 (4)
C1—C7	1.494 (3)	C10—C11	1.400 (4)
C2—C3	1.382 (3)	C11—C12	1.376 (4)
C2—H2	0.9500	C11—H11	0.9500
C3—C4	1.387 (4)	C12—C13	1.380 (4)
C3—H3	0.9500	C12—H12	0.9500
C4—C5	1.375 (4)	C13—C14	1.381 (3)
C4—H4	0.9500	C14—C15	1.390 (4)
C5—C6	1.387 (4)	C14—H14	0.9500
C5—H5	0.9500	C15—H15	0.9500
			
C9—O1—H1	109.5	C7—C8—H8	115.8
C6—C1—C2	119.2 (2)	C10—C8—H8	115.8
C6—C1—C7	121.4 (2)	O2—C9—O1	122.5 (2)
C2—C1—C7	119.4 (2)	O2—C9—C7	121.7 (2)
C3—C2—C1	120.5 (2)	O1—C9—C7	115.8 (2)
C3—C2—H2	119.8	C15—C10—C11	117.5 (2)
C1—C2—H2	119.8	C15—C10—C8	119.7 (2)
C2—C3—C4	119.7 (2)	C11—C10—C8	122.7 (2)
C2—C3—H3	120.1	C12—C11—C10	121.4 (2)
C4—C3—H3	120.1	C12—C11—H11	119.3
C5—C4—C3	120.1 (2)	C10—C11—H11	119.3
C5—C4—H4	120.0	C11—C12—C13	119.4 (3)
C3—C4—H4	120.0	C11—C12—H12	120.3
C4—C5—C6	120.5 (2)	C13—C12—H12	120.3
C4—C5—H5	119.7	C14—C13—C12	121.4 (2)
C6—C5—H5	119.7	C14—C13—Cl1	119.59 (19)
C1—C6—C5	120.0 (2)	C12—C13—Cl1	119.0 (2)
C1—C6—H6	120.0	C13—C14—C15	118.4 (2)
C5—C6—H6	120.0	C13—C14—H14	120.8
C8—C7—C9	120.1 (2)	C15—C14—H14	120.8
C8—C7—C1	124.6 (2)	C10—C15—C14	121.9 (2)
C9—C7—C1	115.3 (2)	C10—C15—H15	119.1
C7—C8—C10	128.4 (2)	C14—C15—H15	119.1
			
C6—C1—C2—C3	0.2 (3)	C1—C7—C9—O2	−3.6 (3)
C7—C1—C2—C3	179.7 (2)	C8—C7—C9—O1	−1.8 (3)
C1—C2—C3—C4	1.2 (4)	C1—C7—C9—O1	178.0 (2)
C2—C3—C4—C5	−1.2 (4)	C7—C8—C10—C15	−155.2 (3)
C3—C4—C5—C6	0.0 (4)	C7—C8—C10—C11	28.7 (4)
C2—C1—C6—C5	−1.4 (3)	C15—C10—C11—C12	1.0 (3)
C7—C1—C6—C5	179.0 (2)	C8—C10—C11—C12	177.2 (2)
C4—C5—C6—C1	1.3 (4)	C10—C11—C12—C13	0.4 (4)
C6—C1—C7—C8	−113.7 (3)	C11—C12—C13—C14	−1.1 (4)
C2—C1—C7—C8	66.7 (3)	C11—C12—C13—Cl1	178.89 (19)
C6—C1—C7—C9	66.6 (3)	C12—C13—C14—C15	0.4 (4)
C2—C1—C7—C9	−113.0 (3)	Cl1—C13—C14—C15	−179.57 (18)
C9—C7—C8—C10	−172.1 (2)	C11—C10—C15—C14	−1.7 (3)
C1—C7—C8—C10	8.1 (4)	C8—C10—C15—C14	−178.1 (2)
C8—C7—C9—O2	176.7 (2)	C13—C14—C15—C10	1.0 (4)

### Hydrogen-bond geometry (Å, °)

**Table:**

*D*—H···*A*	*D*—H	H···*A*	*D*···*A*	*D*—H···*A*
O1—H1···O2^i^	0.84	1.82	2.658 (3)	177

Symmetry codes: (i) −*x*, −*y*−1, −*z*.
